# Phenotypic and Functional Heterogeneity of Low-Density and High-Density Human Lung Macrophages

**DOI:** 10.3390/biomedicines9050505

**Published:** 2021-05-04

**Authors:** Barbara Balestrieri, Francescopaolo Granata, Stefania Loffredo, Angelica Petraroli, Giulia Scalia, Paolo Morabito, Chiara Cardamone, Gilda Varricchi, Massimo Triggiani

**Affiliations:** 1 Department of Translational Medical Sciences, University of Naples Federico II, 80131 Naples, Italy; stefanialoffredo@hotmail.com (S.L.); petrarol@unina.it (A.P.); gildanet@gmail.com (G.V.); 2 Center of Excellence, World Allergy Organization (WAO), 80131 Naples, Italy; 3 Center for Basic and Clinical Immunology Research (CISI), University of Naples Federico II, 80131 Naples, Italy; 4 Institute of Experimental Endocrinology and Oncology (IEOS), National Research Council, 80131 Naples, Italy; 5 Clinical and Experimental Cytometry Unit, CEINGE-Biotecnologie Avanzate, 80131 Naples, Italy; scalia@ceinge.unina.it; 6 Laboratory of Clinical Pathology, A. Cardarelli Hospital, 80131 Naples, Italy; morabito.paolo@fastwebnet.it; 7 Division of Allergy and Clinical Immunology, University of Salerno, 84084 Fisciano, Italy; ch.cardamone@gmail.com (C.C.); mtriggiani@unisa.it (M.T.)

**Keywords:** cancer, IL-6, IL-10, IL-12, lipopolysaccharide, lung, monocytes, macrophages, TNF-α

## Abstract

Background: Pulmonary macrophages are a highly heterogeneous cell population distributed in different lung compartments. Methods: We separated two subpopulations of macrophages from human lung parenchyma according to flotation over density gradients. Results: Two-thirds 65.4% of the lung macrophages have a density between 1.065 and 1.078 (high-density macrophages: HDMs), and the remaining one-third (34.6) had a density between 1.039 and 1.052 (low-density macrophages: LDMs). LDMs had a larger area (691 vs. 462 μm^2^) and cell perimeter (94 vs. 77 μm) compared to HDMs. A significantly higher percentage of HDMs expressed CD40, CD45, and CD86 compared to LDMs. In contrast, a higher percentage of LDMs expressed the activation markers CD63 and CD64. The release of TNF-α, IL-6, IL-10 and IL-12 induced by lipopolysaccharide (LPS) was significantly higher in HDMs than in LDMs. Conclusion: The human lung contains two subpopulations of macrophages that differ in buoyancy, morphometric parameters, surface marker expression and response to LPS. These subpopulations of macrophages probably play distinct roles in lung inflammation and immune responses.

## 1. Introduction

Macrophages are important resident immune cells in all tissues [1], where they play pivotal roles in tissue homeostasis [2,3,4]. In this context, macrophages are critical sentinels in immunity, combating infections, modulating angiogenesis and lymphangiogenesis [5,6,7], resolving inflammation [8,9,10], and surveilling against tumors [4,11,12,13]. Macrophages are implicated in a wide spectrum of disorders, including pulmonary [14] and cardiovascular diseases [15,16], diabetes [17] and cancer [18,19,20,21].

Although macrophages resident in different tissues share several common features and functions, this cell population is highly heterogeneous. Although macrophages can be distinguished by their expression of surface antigens [4,22,23,24], other characteristics help differentiate macrophage subpopulations in mice and humans, including cell-intrinsic density [25,26], and ultrastructural [27] and functional properties [22]. Tissue macrophages arise from distinct cell lineages emerging during embryonic development [28,29,30,31]. In the lung, tissue-resident macrophages homing during embryogenesis self-renew throughout life without replacement from circulating monocytes [32,33]. However, during inflammation, bone marrow-derived monocytes can invade various organs including the lung and locally differentiate into macrophages [34]. Macrophages display a specific phenotype in response to microenvironmental signals. In vitro, two extreme forms of differentiation/activation are generally referred to as “classical” (or M1) and “alternative” (or M2) stage of activation [4,21]. However, in vivo, macrophages exist in a continuous spectrum of activation [35]. Recently, several groups have provided deeper insights into macrophage heterogeneity through single-cell transcriptomics of human [36,37,38,39] and mouse macrophages [37,40].

Pulmonary macrophages constitute a heterogeneous cell population localized in distinct compartments [38,39]. Alveolar macrophages are found in the alveolar walls, whereas interstitial macrophages represent the majority of immune cells in the lung parenchyma [41]. Differences in morphology, phenotype and function between alveolar and interstitial macrophages in rodents [42,43] and in humans [22,27] have been reported. There is also preliminary evidence of phenotypic and functional heterogeneity of human alveolar macrophages [44,45].

Separation of immune cells by density gradients has been used to isolate different subpopulations of rodent macrophages [25,26] and of human mast cells [46], basophils [47], eosinophils [48,49], neutrophils [50,51], and monocytes [52,53]. The identification of cell populations by density has led to the definition of distinct cell subpopulations with specific functions [50,51]. The presence of subpopulations of human lung macrophages with different densities and their functional heterogeneity have not been systematically evaluated. In this study, we have started to characterize two distinct subpopulations of macrophages from human lung parenchyma that differ by density, size, immunological and morphometric features as well as their functional response to lipopolysaccharide (LPS).

## 2. Materials and Methods

### 2.1. Reagents and Buffers

LPS (*E. coli* serotype 026:B6), Percoll, density marker beads (range 1.018–1.098), l-glutamine, antibiotic-antimycotic solution (10,000 UI/mL penicillin, 10 mg/mL strepto-mycin, and 25 µg/mL amphotericin B), Triton X-100, phenolphthalein glucuronide, BSA and PIPES were purchased from Sigma (St. Louis, MO, USA). RPMI and FCS were from ICN (Costa Mesa, CA, USA). IFN-γ was from PeproTech (London, UK). FITC-conjugated anti-CD4 and anti-CD15 mAbs were from Ortho (Amsterdam, The Netherlands). FITC-conjugated anti-CD1a, CD14, CD45, CD69, CD44 and PE-conjugated anti-CD95, CD11c, CD56, CD130, CD154 and HLA-DR mAbs were purchased from Becton Dickinson (San Jose, CA, USA). PE-conjugated anti-CD42b and FITC-conjugated anti-CD61 mAb were from DAKO (Glostrup, Denmark), FITC-conjugated IgG1, anti-CD63, CD38, CD71 and PE-conjugated anti-CD25 mAbs were from Immunotech (Marseille, France). FITC-conjugated anti-CD35 and PE-conjugated anti-CD117 mAbs were from Southern Biotechnology Associates (Birmingham, AL, USA). FITC-conjugated anti-CD64 and PE-conjugated IgG2, and anti-CD86 mAbs were purchased from Caltag (Burlingame, CA, USA). FITC-conjugated anti-CD80 was from Serotech (Oxford, UK). PE-conjugated anti-CD40 mAb was from Ancel (Bayport, MN, USA). Anti-CD206 was from Miltenyi Biotec (Bergisch Gladbach, Germany). TLR-4, MD-2 and MyD88 primers were prepared as described below and purchased from Invitrogen (Rodano, Italy). Rabbit anti-phospho-ERK1/2 Ab, rabbit anti-phospho-p38 Ab and rabbit anti-ERK1/2 Ab were from Cell Signaling (New England Biolabs, Beverly, MA, USA). Rabbit anti-p38 (C-20) Ab was from Santa Cruz (Santa Cruz, CA, USA). HRP-conjugated donkey anti-rabbit Ig Ab was from Amersham Biosciences (Buckinghamshire, UK). All other reagents were from Carlo Erba (Milan, Italy). Piperazine-1,4-bis (2-ethanesulfonic acid) (PIPES) buffer was composed of 25 mM PIPES, 110 mM NaCl and 5 mM KCl, pH 7.4 [54]. PCG buffer was made of PIPES buffer containing 1 mM CaCl_2_ and 1 g/L glucose, pH 7.4. Glycine buffer was composed of 400 mM glycine and 400 mM NaCl, pH 10.3 [55,56]. Lysis buffer (LB) for Western blot was made of 20 mM Tris pH 7.5, 5 mM EDTA, 1 mM PMSF, 2 mM benzamidine, 10 μg/mL aprotinin, 10 μg/mL leupeptin, 10 mM NaF, 150 mM NaCl, 1 mM Na_3_VO_4_, 1% Nonidet P-40 and 5% glycerol [56].

### 2.2. Isolation and Purification of Human Lung Macrophages

The study was approved by the Ethics Committee (25 February 2019) of the University of Naples Federico II (Protocol N. 7/19). Freshly resected lung tissue (approximately 40 grams) was obtained intraoperatively from patients undergoing lobectomy for focal lung tumors. Macroscopically normal lung tissues were obtained from uninvolved regions [6,57]. Lung tissue was minced finely with scissors and washed extensively with PIPES buffer over Nytex cloth (120-μm pore size) (Tetko, Elmsford, NY, USA). Macrophage fractionation by density was accomplished by centrifugation over discontinuous Percoll gradients. Cells were layered onto 25 mL gradients containing 5 mL of different Percoll dilutions in PIPES with densities of 1.026, 1.039, 1.052, 1.065 and 1.078. Cells were centrifuged for 20 min (1000 g) at 22 °C. The cells localized at each interface were then harvested and washed. At the end of this procedure, two fractions of macrophages were obtained: low-density macrophages (LDMs), floating between density bands 1.039 and 1.052, and high-density macrophages (HDMs), floating between density bands 1.065 and 1.078. Each cell fraction was washed and resuspended (10^6^ cells/mL) in RPMI containing 5% FCS, 2 mM L-glutamine, and 1% antibiotic-antimycotic solution. Cells were then incubated in 24-well plates (Falcon Becton Dickinson) (Franklin Lakes, NJ, USA) at 37 °C in an atmosphere of 5% CO_2_ and 95% air. After 12 h, the medium was removed, and the plates were gently washed three times with RPMI. More than 94% of adherent cells were macrophages. All experiments were repeated with cells obtained from different patients.

### 2.3. Light Microscopy and Morphometric Analysis

Cytospins of LDMs and HDMs were prepared by centrifugation of 20,000 cells onto microscope slides using a Shandon Cytospin 3 Cytocentrifuge (Shandon Astmoor, UK). Slides were allowed to air dry and stained with Diff-Quick (Biomap, London, UK). Cell morphology was evaluated by light microscopy focusing on the homogeneity in each macrophage subpopulation and differences between LDMs and HDMs. For morphometric analysis, cells were measured from randomly chosen microscope fields with a Zeiss Kontron Videoplan Analysis system coupled with a Zeiss microscope (630×) and a Grundig FA 184 electronic black and white camera. This device reproduces the cellular images in a monitor. Measurements were made as previously described [58].

### 2.4. Flow Cytometry

**Figure 1:**
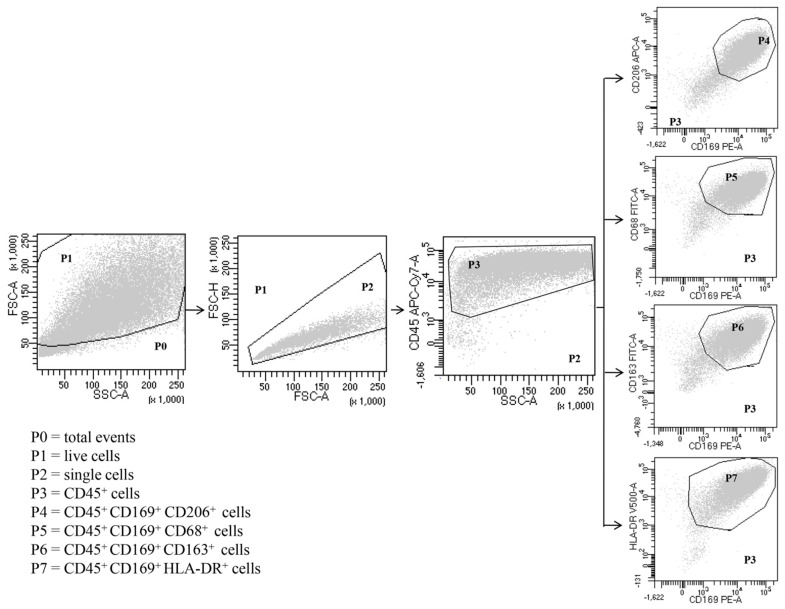
Representative flow cytometric panels with respect to the gating strategy used to identify human lung macrophages. Among total cells (P0), after excluding dead cells (P1) and doublets (P2), leukocytes were identified as CD45^+^ (P3). Macrophages were identified as SSC^high^ FSC^high^ CD45^+^ CD169^+^ (P4-P7) CD206^+^ (P4) CD68^+^ (P5) CD163^+^ (P6) HLA-DR^+^ (P7). Results are representative of three independent experiments.

Expression of surface markers in total HLM, LDM and HDM was analyzed by direct immunofluorescence and flow cytometry (FACSCalibur or FACS-Canto II) (Becton Dickinson, Milan, Italy), according to the following protocol. Freshly isolated macrophages were suspended in PBS at a concentration of 5 × 10^6^ cells/mL. Fifty microliters of cell suspension were incubated (4 °C, 20 min in the dark) with saturating amounts of antibodies (Abs). Erythrocytes were lysed in 2 mL of BD lysis buffer (BD Bioscience, Milan, Italy) (22 °C, 20 min). To quench high spontaneous autofluorescence of human lung macrophages (HLMs), cells were washed twice with PBS, suspended in 0.2 mL of saturated Crystal Violet solution (Certistain, Merck, Darmstadt, Germany) and incubated for 5 min at 22 °C [59]. The Cell-Quest software (Becton Dickinson, Milan, Italy) was used for acquisition and analysis according to a dual color procedure. In other experiments, the cells, obtained after Percoll gradient centrifugation, were suspended (10^6^ cells/mL) in RPMI 1640 with 5% FCS, 2 mM l-glutamine, and 1% antibiotic-antimycotic solution and incubated in 24-well plates. After 12 h, the medium was removed, and the plates were gently washed with fresh medium. More than 94% of adherent cells were macrophages, as evaluated by flow cytometry with the following Abs: anti-CD68 FITC, anti-CD163 FITC, anti-169 PE, anti-CD206 APC, anti-CD24 HV 450 (Miltenyi Biotec, Bergisch Gladbach, Germany), anti-CD5 PE, anti-CD123 APC, HLA-DR HV500, anti-CD22 APC (Becton Dickinson, Milan, Italy), anti-CD14 PE-Cy7 (Life-technologies, Monza, Italy) and anti-CD45 APC-Cy7 (BioLegend, London, UK). Adherent lung cells were examined initially by forward scatter (FSC) area versus side scatter (SSC) area and then by FSC area versus FSC height, with gating on single cells to eliminate dead cells, debris and clumped cells from the analysis (Figure 1). Single cells were then examined by CD45 expression, gating on CD45^+^ cells, which represented total leukocytes. As expected, the majority of the adherent lung cells were CD45^+^ leukocytes. Within these cells, CD169 (siglec-1), CD206 (mannose receptors), CD68, CD163 and HLA-DR were used to identify macrophages as previously described [39]. The vast majority of CD169^+^ cells were human lung macrophages, which were CD206^+^, CD68^+^, CD163^+^ and HLA-DR^+^ (Figure 1). The remaining CD45^+^ cells (Supplementary Figure S1) from gate P3 were examined by (1) SSC-A versus CD14 to distinguish CD14^high^ cells (P4), which represent essentially monocytes (0.3%); (2) SSC-A versus CD22 to identify CD22^high^ cells (P5) that are B lymphocytes (0.4%); (3) SSC-A versus CD5 to identify CD5^high^ cells (P6), which represent T lymphocytes (1.2%) (Supplementary Figure S1). The monocytes CD45^+^ CD14^high^ were also identified as CD169^−^ CD206^−^ CD68^+^ CD163^+^ (Supplementary Figure S2). In CD45^+^ cells, we examined the presence of granulocytes by CD24 versus CD169 expression. The cell population CD24^+^ CD169^−^ represents the granulocytes (4%) (Supplementary Figure S3A). To evaluate the dendritic cell (DC) contamination, we searched the presence of CD169^−^ CD123^+^ cells. DCs were not found in human lung macrophage preparations because all CD169^−^ CD123^+^ cells were monocytes, being also CD14^high^ (0.4%) (Supplementary Figure S2B). The samples were acquired by FACS-Canto II and analyzed by FACS-DiVa software (Becton Dickinson, Milan, Italy). Values were expressed as the percentage of positive and negative cells. The cut-off point between positive and negative cells was set using control antibodies of the same isotype. Cells were scored positive when their fluorescence intensity was greater than that observed on 99% of cells stained with negative controls. Fluorescence intensity was also analyzed by recording the mean fluorescence intensity (MFI) expressed in linear units. Background fluorescence was assessed by analyzing isotypic control antibodies.

### 2.5. Cell Incubations

Human lung macrophages adherent to 24-well plates were incubated (37 °C, 6–24 h) in RPMI containing increasing concentrations of LPS (0.1 to 10 μg/mL). In the experiments evaluating IL-12 production, cells were preincubated (37 °C, 1 h) with IFN-γ (1000 U/mL) and then stimulated with LPS. At the end of incubation, the supernatant was removed, centrifuged twice (1000 g, 4 °C, 5 min) and stored for up to 72 h at −80 °C for subsequent determination of IL-6, TNF-α, IL-10 and IL-12 release. After each experiment, an aliquot of cells was stained with Trypan blue to determine cell viability. The cells remaining in the plates were lysed with 0.1% Triton X-100 for the determination of total cell protein content.

### 2.6. Protein and β-Glucuronidase Assays

The total content of cell proteins [60] and β-glucuronidase [55] was determined as previously described in cell aliquots lysed with 0.1% Triton X-100. The standard curve of β-glucuronidase activity was prepared using, as standard, β-glucuronidase from *Helix pomatia* (Sigma, St. Louis, MO, USA).

### 2.7. ELISA Assays

IL-6, TNF-α, IL-10 and IL-12 concentrations in the macrophage culture supernatant were measured in duplicate by ELISA (Euro Clone, Torquay, UK), according to the manufacturer’s instructions. The linearity ranges of the assays were between 6.25 and 200 pg/mL (IL-6), 25 and 800 pg/mL (TNF-α), 12.5 and 400 pg/mL (IL-10), 6.25 to 200 pg/mL (IL-12) [61]. Since the number of adherent macrophages can vary in each well and in different experiments, the results were normalized for the total protein content of each sample.

### 2.8. Isolation of Cellular Mrna and RT-PCR

Total RNA from human lung macrophages was extracted using the SV 96 total RNA isolation system (Promega, Milan, Italy) according to the manufacturer’s instructions, and treated with Rnase-free DNase I. Reverse transcription was performed with 5 mM MgCl_2_, oligod(t)16 primer, and MuLV reverse transcriptase according to the manufacturer’s instructions (Applied Biosystems, Norwalk, CT, USA) on a thermocycler (GeneAmp PCR System 1400; Applied Biosystems, Norwalk, CT, USA). cDNA was then titrated for β-actin message [61], and equivalent templates of cDNAs, were amplified for TLR4 (isoforms 1-3) and TLR4 (isoform 2) using the specific primers designed according to the published sequences (GenBank accession n. NM_138554 for isoforms 1-3 and NM_138556 for isoform 2). Primers sequences were: TLR4 (isoforms 1-3) forward primer 5′-AGA ACT GCA GGT GCT GGA TT-3′ and reverse primer 5′-AAA CTC TGG ATG GGG TTT CC-3′ and TLR4 (Isoform 2) forward primer 5′- AGT GAG GAT GAT GCC AGG AT-3′ and reverse primer 5′-TTC ATG CCA GCT CTT CTG TG-3′. The primers specific for MyD88 and MD-2 were prepared as reported. The amplification protocol consisted of 30 cycles as follows: denaturation, 1 min 95 °C; annealing, 1 min at 54 °C for TLR4 (isoforms 1-3 and 2), 55 °C for MyD88 and 56 °C for MD-2; and 2 min primer extension at 72 °C. A final extension at 72 °C for 10 min was performed. The PCR products, together with a DNA ladder as a size standard, were separated on 2% agarose gel, stained with ethidium bromide and photographed. Real-time quantitative PCR was performed using the iCycler (Bio-Rad Hercules, CA, USA) SYBR Green fluorophore core reagent kit (PE Applied Biosystems Foster City, CA, USA) and TLR4 (isoforms 1 and 3) and TLR4 (isoform 2) primers described above.

### 2.9. Phosphorylation of ERK1/2 and p38 Kinases

Phosphorylation of MAP kinases was assessed as previously described [56]. Purified macrophages were suspended in PCG buffer. Cells (10^6^ per sample) were incubated (37 °C, 1–120 min) with 1 μg/mL LPS. At the end of incubation, the reactions were stopped by adding ice-cold PIPES buffer and samples were microfuged for 30 s. Cell pellets were immediately lysed in lysis buffer (LB). Cell lysates were kept on ice for 20 min and then microfuged for 15 min at 4 °C. The supernatant was collected as a protein extract containing lysed cell components without nuclei and diluted in electrophoresis sample buffer (ESB, Novex, Invitrogen) containing 5% 2-mercaptoethanol (2-ME). Proteins were separated on 10% Bis-Tris gels (NuPAGE^®^, Novex, Milan, Italy) and transferred to a nitrocellulose membrane (Biorad, Hercules, CA, USA). After immersion overnight in TBST (50 mM Tris p H 7.5, 150 mM NaCl and 0.05% Tween 20) containing 4% BSA, the membranes were washed three times (10 min each) with TBST and then blotted (22 °C, 2 h) with anti-phospho ERK1/2 Ab or anti-phospo-p38 Ab. After washing, the membranes were incubated (22 °C, 1 h) with HRP-conjugated anti rabbit Ig Ab. Membranes were rinsed four times and membrane-bound anti-rabbit Ig Ab was visualized with ECL western blotting detection reagents (Amersham Biosciences) (Milan, Italy) and HyperECL luminescence detection film (Amersham Bioscience) (Milan, Italy). Although comparisons were made on the basis of an equal number of cells, the membranes were stripped (24 h, 4 °C) with stripping buffer (7 M guanidine hydro-chloride in distilled water) and reblotted with anti-ERK1/2 Ab or anti-p38 Ab to assess the equal protein content of each sample.

### 2.10. Statistical Analysis

Data were analyzed with the GraphPad Prism 6 software package (Graphpad Software, La Jolla, CA, USA). Values are expressed as the mean ± SEM (standard error of the mean). Statistical analysis was performed using Student’s *t*-test for unpaired samples. A *p* value < 0.05 was considered statistically significant.

## 3. Results

### 3.1. Morphometrical and Biochemical Characteristics of Human Lung Macrophage Subpopulations

**Figure 2:**
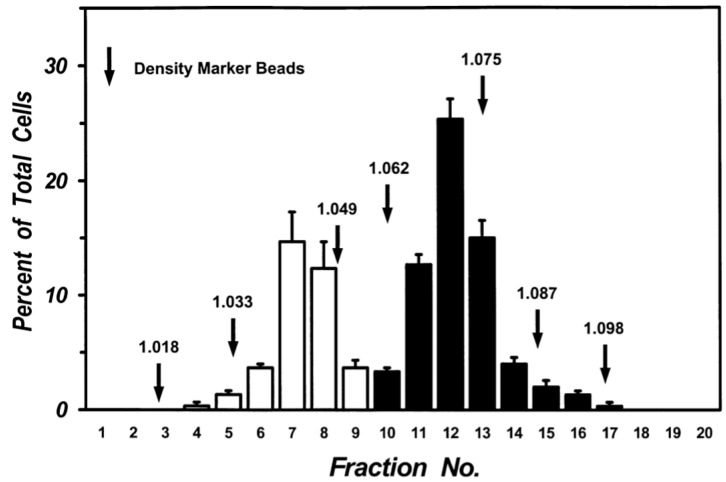
Separation of human lung macrophages by continuous density gradient. Cells (10^7^) were layered on continuous Percoll gradient prepared as described in Materials and Methods and centrifuged (1000 *g*, 22 °C, 20 min). Density marker beads were layered and centrifuged in a separate tube. Fractions of 0.5 mL were collected and the cells in each fraction were counted. Data are the mean ± SEM of four experiments.

Mechanical dispersion of macroscopically normal human lung tissue yielded a cell preparation containing 5.63 ± 1.01 × 10^6^ macrophages per gram of tissue with a purity of 65.4 ± 2.3% (*n* = 10). Centrifugation of lung macrophages over discontinuous density gradients yielded two sets of cells at the interface between density bands of 1.039 and 1.052 (Low-Density Macrophages: LDMs) and the interface between 1.065 and 1.078 (High-Density Macrophages: HDMs). A clear separation of the two sets of cells was consistently obtained in multiple preparations from twenty donors. The two cell subpopulations were enriched in macrophages (purity between 85% and 97%). The macrophages in the two subpopulations were further purified (95–98% purity) by additional discontinuous gradients after which each population retained its own density characteristics. Figure 2 shows the data from five different lung preparations. There was a clear bimodal distribution of macrophages: one subpopulation had a density between 1.033 and 1.049 (white columns) whereas another had a density between 1.062 and 1.087 (black columns). Each population retained its density migration after incubation (37 °C, 24 h) indicating that the density of each macrophage subpopulation is a stable characteristic, which is maintained during short-term culture (data not shown).

**Figure 3:**
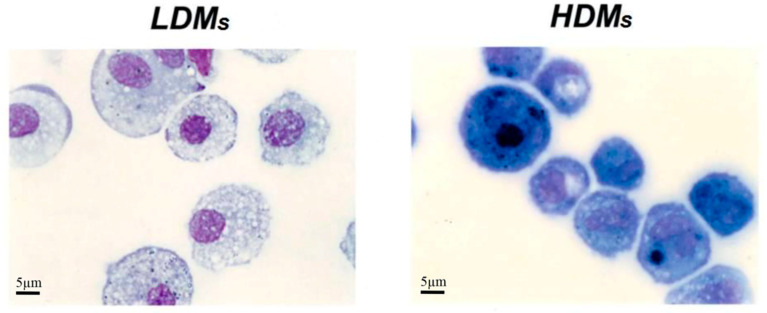
Cytocentrifuge preparation of HDMs and LDMs isolated by density gradient and adherence. Cell were stained with Diff Quick and analyzed by light microscopy. LDMs have round nuclei and an extended cytoplasm that stains poorly and is largely occupied by vacuoles. HDMs mostly comprise smaller cells with horse-shoe shaped nuclei; the cytoplasm is strongly basophilic and sometimes exhibits a juxtanuclear Golgi area.

Table 1 reports basic morphometric and biochemical parameters of the two macrophage subpopulations. HDMs constitute the majority (65.4 ± 6.5%) of the macrophages retrieved by mechanical disruption of the lung tissue, whereas LDMs account for the remaining 34.6 ± 6.5%. LDMs have a larger cell area (691.27 ± 20.49 μm^2^ vs. 462.62 ± 11.75 μm^2^; *p* < 0.001) and perimeter (94.44 ± 1.35 μm vs. 76.97 ± 0.94 μm; *p* < 0.05) than HDMs. The two subpopulations of lung macrophages have a similar content of total cell protein and of the lysosomal enzyme β-glucuronidase (Table 1). Light microscopy analysis showed that the two macrophage subpopulations are morphologically different. LDMs have round nuclei and an extended cytoplasm that stains poorly and is largely occupied by vacuoles. HDMs mostly comprise smaller cells with horse-shoe shaped nuclei; the cytoplasm is strongly basophilic and sometimes exhibits a clear juxtanuclear Golgi area. Carbon inclusions are evident in some cells of both preparations (Figure 3).

### 3.2. Phenotypic Characterization of Human Lung Macrophage Subpopulations

In six experiments, we examined the expression of several surface molecules in the two macrophage subpopulations separated by density gradients. Table 2 reports markers expressed on more than 85% or on less than 5% of the cells in both subpopulations. The major markers of tissue macrophages (CD206, CD11c, CD44, CD71, HLA-DR) were highly expressed on both LDMs and HDMs. The low expression (<5%) of CD1a (dendritic cells), CD4 (T cells), CD56 (NK cells), CD69 (eosinophils) and CD117 (mast cells) indicated that contaminating cells in HDM and LDM preparations are negligible.

**Figure 4:**
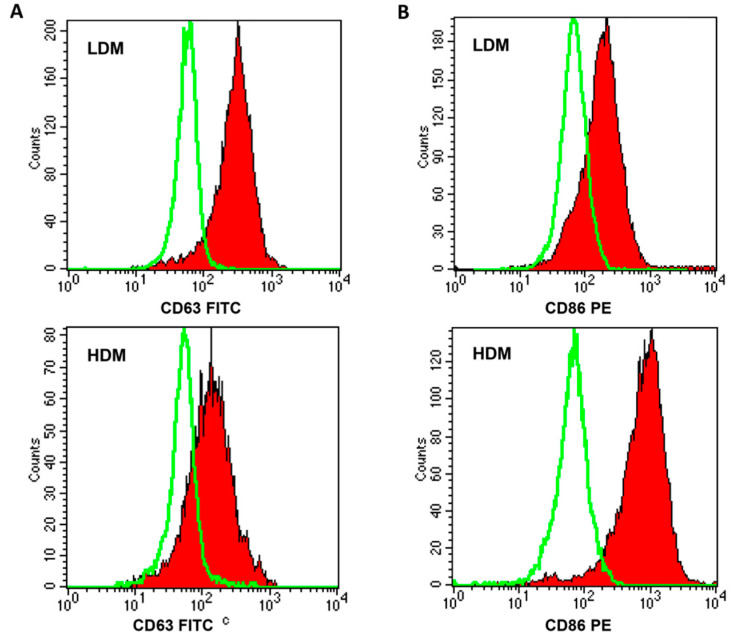
FACS analysis of CD63 and CD86 expression on LDMs and HDMs. Freshly purified lung macrophages from the two subpopulations were suspended in PBS at a concentration of 5 × 10^6^/mL. Fifty microliters of cell suspension were incubated (4 °C, 20 min) with saturating amounts of FITC-conjugated anti-CD63 (Panel (**A**), closed histogram) or PE-conjugated anti-CD86 (Panel (**B**), closed histogram) Abs. Background fluorescence (Panels (**A**) and (**B**), open histogram) was assessed using isotypic control Abs. Quenching of macrophage autofluorescence and flow cytometry analysis were performed as described in Materials and Methods. The figure shows the results of one representative experiment of the six performed.

Next, we wanted to ascertain whether the two subpopulations showed a differential expression of relevant surface markers (Table 3). In six different preparations of macrophages, a higher percentage of HDMs expressed CD40, CD45, and CD86 as compared to LDMs, whereas larger proportions of CD63^+^ and CD64^+^ cells were found in LDMs. Figure 4 shows representative flow cytometry scans illustrating the differential expression of CD63 (Panel A), mostly found on LDMs, and of CD86 (Panel B), predominantly expressed on HDMs. These results indicate that the expression of some surface markers differ quantitatively between LDMs and HDMs.

### 3.3. Release of Cytokines from Human Lung Macrophage Subpopulations Induced by LPS

**Figure 5:**
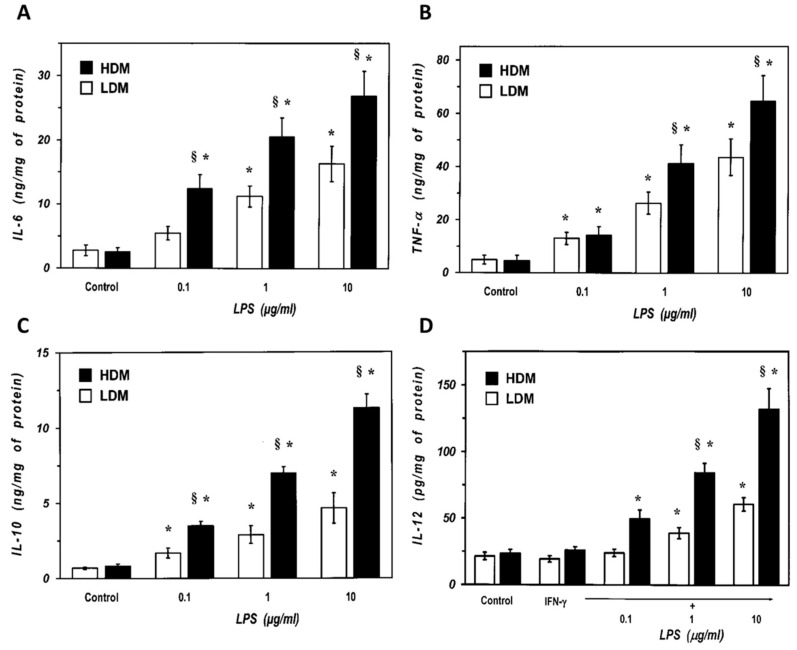
Effects of increasing concentrations of LPS on the release of IL-6 (**Panel A**) and TNF-α (**Panel B**) from LDMs and HDMs. Cells were incubated (37 °C, 24 h) with the indicated concentrations of LPS. The supernatants were collected and centrifuged (1000 g, 4 °C, 5 min). IL-6 and TNF-α were determined by ELISA. Values are expressed as ng of cytokine/mg of protein. Data are the mean ± SEM of four experiments. * *p* < 0.05 vs. respective control, ^§^ *p* < 0.05 vs. LDMs. (**Panel C**) Effects of increasing concentration of LPS on the release of IL-10 from LDMs and HDMs. Cells were incubated (37 °C, 24 h) with the indicated concentrations of LPS. The supernatants were collected and centrifuged (1000 *g*, 4 °C, 5 min). IL-10 was determined by ELISA. Values are expressed as ng of IL-10/mg of protein. Data are the mean ± SEM of four experiments. * *p* < 0.05 vs. respective control, ^§^ *p* < 0.05 vs. LDMs. (**Panel D**) Effects of increasing concentrations of LPS on the release of IL-12 from LDMs and HDMs. Cells were preincubated (37 °C, 1 h) with IFN-γ (1000 U/mL) and stimulated (37 °C, 24 h) with the indicated concentrations of LPS. The supernatants were collected and centrifuged (1000 *g*, 4 °C, 5 min). IL-12 was determined by ELISA. Values are expressed as pg of IL-12/mg of protein. Data are the mean ± SEM of four experiments. * *p* < 0.05 vs. respective control, ^§^ *p* < 0.05 vs. LDMs.

We next examined the response of the two subpopulations of lung macrophages to LPS, the main proinflammatory component of Gram negative bacteria and a potent inducer of cytokine production by lung macrophages [6]. Macrophages were stimulated with LPS (0.1 to 10 µg/mL), and the release of proinflammatory (TNF-α, IL-6) and immunomodulatory (IL-10 and IL-12) cytokines was determined. Figure 5 shows that there was no difference in the spontaneous production of IL-6 and TNF-α between LDMs and HDMs (Panel A and B). LPS (0.1–10 μg/mL) induced a concentration-dependent release of IL-6 from both LDMs and HDMs (Figure 5A). However, all concentrations of LPS induced an almost two-fold release of IL-6 from HDMs than from LDMs. Similarly, LPS was also more effective in inducing TNF-α release from HDMs than from LDMs (Figure 5B). IL-10 and IL-12 are produced by lung macrophages and are involved in the regulation of immune responses [62,63]. Figure 5C shows that there was no difference in the spontaneous release of IL-10 between LDMs and HDMs. LPS (0.1–10 μg/mL) stimulated the release of IL-10 in a concentration-dependent fashion from both HDMs and LDMs. At all concentrations tested, LPS induced a two- to three-fold larger release of IL-10 from HDMs than from LDMs. LPS is an effective stimulus for IL-12 production in lung macrophages only when cells are preincubated with IFN-γ [64]. In addition, in our experimental conditions, human lung macrophages release much lower amounts of IL-12 (in the pg range) than other cytokines (Figure 5D). LPS (0.1–10 μg/mL) induced a concentration-dependent release of IL-12 from HDMs, whereas it was much less effective on LDMs (Figure 5D). Although statistically significant at the highest concentrations of LPS (1 and 10 µg/mL), the net release of IL-12 from LDMs was small and it was not further increased by concentrations of LPS above 10 µg/mL (data not shown).

**Figure 6:**
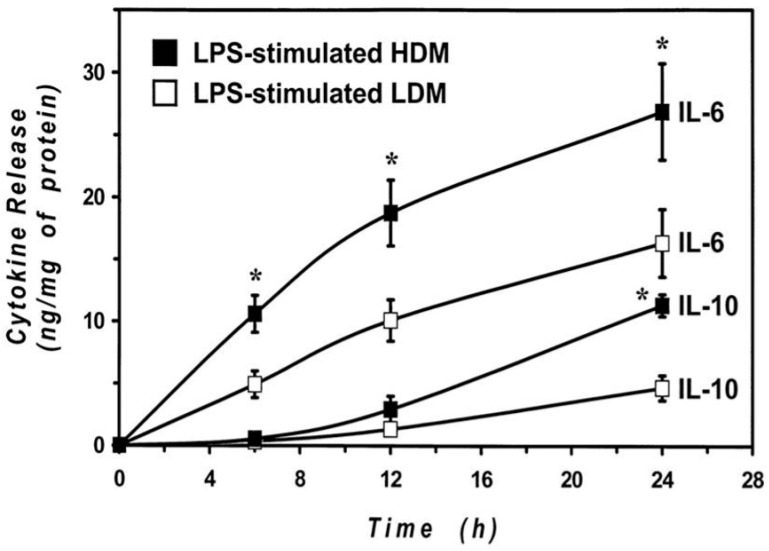
Kinetics of IL-6 and IL-10 release induced by LPS from LDMs and HDMs. HDMs and LDMs were incubated with LPS (1 µg/mL) for different times (6–24 h). Supernatants were collected and centrifuged (1000 g, 4 °C, 5 min). IL-6 and IL-10 release was determined by ELISA. Values are expressed as ng of cytokine/mg of protein. Data are the mean ± SEM of four experiments. * *p* < 0.05 vs. control.

The cytokine release results were obtained by stimulating the cells with LPS for 24 h to allow an appropriate comparison between LDMs and HDMs. Previous results from our group [55,65] and other groups [66,67] indicate that the kinetics of release of proinflammatory cytokines (IL-6 and TNF-α) are faster than those of immunomodulatory cytokines (IL-10 and IL-12). To explore whether differences in the kinetics may account for the observed difference in cytokine release, we examined the time-course of the IL-6 and IL-10 production by HDMs and LDMs. The results illustrated in Figure 6 demonstrate that the release of IL-6 is faster than that of IL-10 in both macrophage subpopulations. In addition, they also show that there was no significant difference in the kinetics of cytokine release between HDMs and LDMs. The difference in the production of IL-6 between LPS-stimulated HDMs and LDMs was already significant after 6 h of incubation, whereas that of IL-10 required 24 h to reach significance. These data indicate that LPS-induced production of cytokines is quantitatively different between HDMs and LDMs and that it is not due to differences in the release kinetics.

### 3.4. Expression of TLR4, MD-2 and MyD88 in Human Lung Macrophage Subpopulations

**Figure 7:**
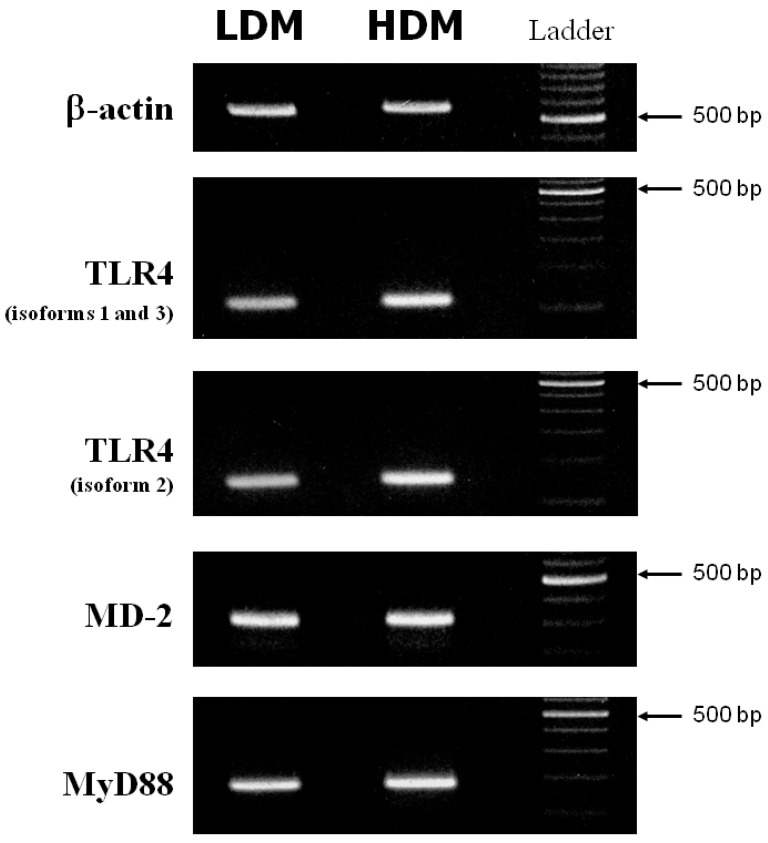
RT-PCR of TLR4, MD-2 and MyD88 from LDMs and HDMs. β-actin, TLR4 (isoforms 1 and 3 = second row; isoform 2 = third row), MD-2 and MyD88 specific RT-PCR amplification products from freshly isolated LDMs and HDMs. This figure shows one representative experiment of the three performed.

Activation of macrophages in response to LPS is mediated by activation of Toll-like receptor 4 (TLR4) [68]. The accessory protein, MD-2, physically associates with TLR4 to form an active TLR4/MD-2 complex that functions as a signaling receptor on the macrophage surface [69]. Main upstream transduction events activated by LPS-TLR4/MD-2 binding also involve the adaptor molecule MyD88 [70]. A defective expression of TLR4 [71,72], MD-2 [69] or MyD88 [73] reduces the macrophage response to LPS. We investigated whether the different responsiveness to LPS of LDMs and HDMs could be due to altered expression of TLR4, MD-2 or MyD88. Figure 7 depicts the β-actin (first row), TLR4 (second and third rows), MD-2 (fourth row) and MyD88 (fifth row) specific RT-PCR amplification products from one experiment representative of three from different preparations of lung macrophages. (Appropriate size mRNAs for TLR4, MD-2 and MyD88 were expressed in both LDMs and HDMs). While no difference was observed in the expression of MD-2 and MyD88 between the two subpopulations, TLR4 may be slightly more expressed in HDMs than in LDMs. To make an accurate comparison of TLR4 expression in the two macrophage subpopulations, we used the real-time quantitative RT-PCR technique to identify the various isoforms of TLR4. In three different preparations of lung macrophages, the relative expression of the various isoforms of TLR4 in HDMs vs. LDMs was 1.74 ± 0.55 (isoforms 1–3) and 1.59 ± 0.39 (isoforms 2), respectively. These values are not statistically different. Together these data indicate that differences in cytokine production between HDMs and LDMs are not due to changes in the expression of TLR4, MD-2 or MyD88.

### 3.5. Activation of p38 and ERK1/2 Kinase in Human Lung Macrophage Subpopulations

**Figure 8:**
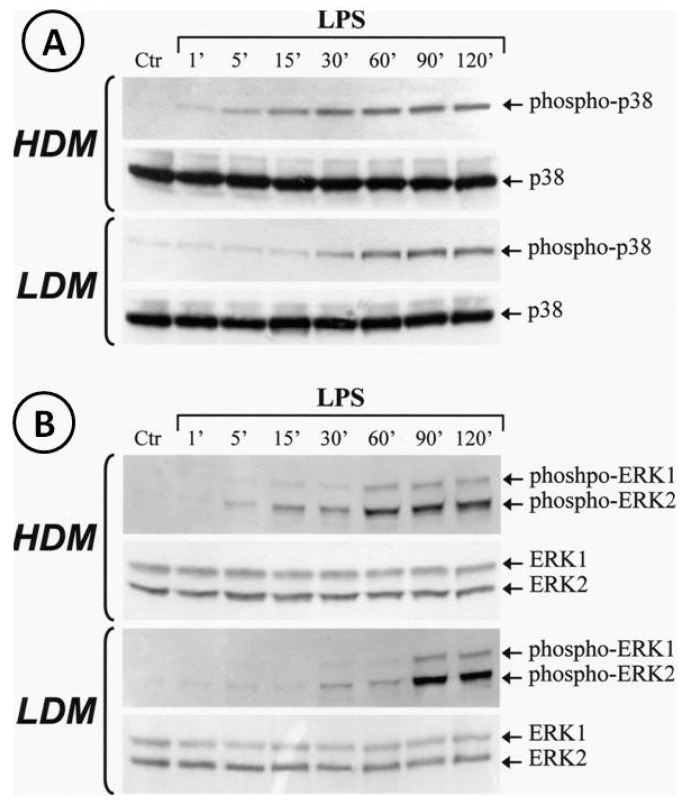
Kinetics of phosphorylation of p38 and ERK1/2 induced by LPS in LDMs and HDMs. Cells were stimulated with LPS (1 µg/mL) for the times indicated. Reactions were stopped by addition of ice-cold PIPES buffer. Cell pellets were microfuged, lysed and subjected to Western blot analysis with anti-phosphorylated-p38 (**Panel A**, gels 1 and 3) or anti-phosphorylated-ERK1/2 (**Panel B**, gel 1 and 3) Abs as described in Materials and Methods. Membranes were then stripped and reblotted with anti-p38 (**Panel A**, gel 2 and 4) or anti-ERK1/2 (**Panel B**, gels 2 and 4) Abs to verify equal protein content in each sample. The Western blot shown is representative of three separate experiments.

Activation of the TLR4/MD-2 complex by LPS leads to the triggering of several transduction pathways [74]. In particular, LPS-induced cytokine gene transcription involves activation of the extracellular signal-regulated kinases (ERK1/2) and p38 stress-activated protein kinase-2 (p38/SAPK2) [74]. To address the hypothesis that LPS may differentially activate ERK1/2 and/or p38 in LDMs and HDMs, we stimulated the two macrophage subpopulations with LPS (1 µg/mL). The reaction was stopped at various time points and cytosolic extracts were subjected to Western blot with anti-phospho-ERK1/2 Ab or with anti-phospho-p38 Ab. Figure 8 shows that LPS induced the phosphorylation of p38 (Panel A) and ERK1/2 (Panel B) in both HDMs and LDMs. However, the kinetics of phosphorylation of these kinases were quite different in the two macrophage subpopulations. In three different macrophage preparations, activation of p38 kinase (Panel A, first gel) in HDMs was already evident after 5 min of incubation and peaked after 30 min, whereas in LDMs (Panel A, third gel), it started after 30 min of incubation and peaked after 60 min. Similarly, peak activation of ERK1/2 kinases occurred after 60 min in HDMs (Panel B, first gel) but only after 90 min in LDMs (Panel B, third gel). Re-probing the membranes with anti-ERK1/2 Ab anti-p38 Ab (non-phosphorylated forms) confirmed equal protein loading (Figure 8, Panel A/B, second and fourth gels). These results indicate that phosphorylation of p38 and ERK1/2 kinases is significantly delayed in LDMs as compared with HDMs.

## 4. Discussion

In this study, we found that two subpopulations of human lung-resident macrophages, separated by buoyancy property, differ by morphometry, phenotype, and functional response to LPS. The density distribution of lung macrophages is clearly bimodal on continuous gradients and it is maintained during short-term culture in vitro.

Pulmonary macrophages are a highly heterogeneous cell population distributed in different compartments. The lung contains macrophages with different origins and functions, including populations of primitive yolk sac-derived macrophages and monocyte-derived macrophages [28,30,31,32]. This ontological classification has several advantages over the canonical M1-M2 classification system [4,21,35]. More recently, single-cell trascriptomics from mouse and human lung have provided deeper insight into human macrophage heterogeneity [36,37,38,39]. In the past, separation of immune cells by density gradients provided some evidence of heterogeneity of rodent macrophages [25,26] and of human mast cells [46], basophils [47] and eosinophils [48,49]. More recently, the identification of low- (LDNs) and normal-density neutrophils (NDMs) highlighted the distinct roles of these two subpopulations in inflammatory disorders [51] and in cancer [50].

Preliminary evidence of density heterogeneity of human lung macrophages has been provided either with BAL cells [44,52] or with cells obtained by enzymatic digestion of lung tissue [22]. In this study, we used cells obtained by gentle mechanical dispersion of human lung parenchyma to avoid possible effects of the harsh enzymatic treatment of lung tissue on the biochemical, phenotypical and functional properties of macrophages.

The two subpopulations (LDMs and HDMs) of human lung macrophages identified in this study express high levels of major macrophage markers (e.g., CD206, CD11c, CD44, CD71, HLA-DR) [39,75,76]. LDMs and HDMs differ in the expression of surface molecules involved in relevant macrophage functions. A higher percentage of LDMs express FcγRI (CD64) and CD63. In contrast, a larger number of macrophages in the HDM population express CD40 and CD86, which are primarily involved in interaction with T cells [77]. Although our observation indicates that LDMs mainly express activation markers (CD63 and CD64), it remains to be investigated whether LDMs and HDMs represent two activation stages of the same cell subset.

Another aim of this study was to explore the responsiveness of the two subpopulations of macrophages to stimulation with LPS. LPS-induced cytokine production from macrophages is involved in many clinical manifestations of several acute and chronic inflammatory disorders [6,34]. Consequent to binding to CD14 on the cell membrane [68], LPS is presented to TLR4, the signaling component of the receptor complex [72] in the presence of protein MD-2 [69]. Our flow cytometry data indicate that both subpopulations of lung macrophages express medium levels of CD14 compared to high levels of CD14 expressed in monocytes. However, both HDMs and LDMs synthesize various cytokines when activated with LPS. This apparently conflicting observation can be explained in several ways. First, there is evidence that CD14-deficient macrophages can be activated by Gram-negative bacteria through activation of CD11b/CD18 and TLR4 [78]. In addition, CD14 deficient mice show significant responses to LPS [79], suggesting that other molecules can compensate for CD14 deficiency. Second, we cannot exclude the possibility that even low expression of CD14 may be sufficient to form an active trimeric TLR4/MD-2/CD14 complex. Finally, a direct interaction of LPS with the cell surface dimeric TLR4/MD-2 complex in the absence of CD14 has been reported [80].

Our study shows that LMDs and HDMs produce different amounts of cytokines upon activation with the same stimulus. In fact, LPS-stimulated HDMs produce significantly higher levels of classical proinflammatory cytokines (i.e., IL-6 and TNF-α) compared to LDMs. This difference is even more marked in the case of the immunoregulatory cytokines IL-10 and IL-12. These observations support the concept that differences in the intensity rather that the quality of the response to LPS is a feature of the two subpopulations of human lung macrophages.

We also investigated several mechanisms that could explain the quantitatively different cytokine production by HDMs and LDMs in response to LPS. Reduced or abolished cytokine synthesis in LPS-activated macrophages has been reported in cells lacking the TLR4 gene [71,72]. In addition, MD-2 [69] and the adapter molecule MyD88 [73] are crucial for an efficient response to LPS. Our results show that there is no difference in the expression of the TLR4 isoforms, MD-2 and MyD88 between HDMs and LDMs. Thus, it is unlikely that changes in the transcription of these molecules may be responsible for the different response to LPS. In contrast, the hyporesponsiveness of LDMs to LPS may be mediated by defective expression of one of the many transducers that are called into play after TLR4 engagement [81]. Downstream signaling activated in LPS-stimulated macrophages includes the MAPK pathways ERK1/2, p38 and JNK [82]. These pathways directly or indirectly activate various nuclear factors involved in the control of cytokine gene expression [74]. Although LPS activated ERK1/2 and p38 in both HDMs and LDMs, the kinetics of activation were different in the two macrophage subpopulations. In fact, phosphorylation of both kinases was delayed in LDMs compared to HDMs. Changes in the time of activation of MAP kinases are associated with different patterns of cytokine production in macrophages [83]. In addition, the time-course of MAP kinase activation in response to the same stimulus depends on maturation stage in various cell lines including monocytes/macrophages [82]. These observations indicate that activation of kinases such as p38 and ERK1/2 is not a stereotypical response since it can be modulated depending on the stimulus used and cell maturation stage. Our results do not prove that the delay of MAP kinase activation is responsible for the lower production of cytokines by LDMs. However, alteration in these signaling pathways could explain, at least in part, the hyporesponsiveness of LDMs to LPS. Although LPS activation reveals only quantitative differences, it is possible that other stimuli may reveal qualitative differences in the response of HDMs and LDMs.

Our study has some limitations that should be pointed out. The in vitro experiments were performed using primary macrophages obtained from macroscopically normal lung parenchyma of patients undergoing thoracic surgery for lung cancer. Although differences between the two cell subpopulations were found in all preparations examined, the possibility that the underlying disease may have affected some of our findings cannot be dismissed. We cannot exclude the possibility that in vivo exposure of human lung macrophages to tumor-derived mediators could explain some of our results. In addition, macrophages, although obtained from macroscopically normal lung tissue, are in close proximity to lung cancer cells. We cannot exclude the possibility that a percentage of LDMs or HDMs are tumor-associated macrophages [19,84]. There is also the possibility that in vivo exposure to altered tumor microenvironment, such as low pH [85], hypoxia [86,87], lactate [88] or adenosine [89] may have affected the phenotypic and the functional activity of lung macrophages. In addition, it has been recently demonstrated that macrophages isolated from lung adenocarcinoma differ phenotypically from those obtained from distant and macroscopically normal lung parenchyma [39]. However, the evidence that HDMs and LDMs differ in their morphometric parameters, phenotype, and response to LPS are in favor of a real distinction between these two subpopulations of macrophages.

The extreme complexity and the clinical exploitation of human lung macrophage heterogeneity is only now being recognized by single-cell RNA seq [36,37,38,39]. Our study indicates that human lung parenchyma contains two subpopulations of macrophages that differ by buoyancy property and quantitative cytokine production. It is unknown whether the cell-intrinsic density of LDMs and HDMs is developmentally acquired or reflects the in vivo plasticity of lung macrophages. We recognize that the full LDM and HDM heterogeneity and differentiation landscape remain incompletely characterized. Further studies, including single-cell trascriptome profiling of LDMs and HDMs, will likely characterize these two subpopulations of human macrophages, and will help to elucidate their functions in different pathophysiological conditions.

## Figures and Tables

**Table 1:** Morphometric and biochemical characteristics of macrophage subpopulations in the human lung.

Parameter	LDMs	HDMs
Density ^a^ (d)	1.039 < d < 1.052	1.065 < d < 1.078
% of total HLM ^a^	34.6 ± 6.5	65.4 ± 6.5
Area^b^ (µm^2^)	691.27 ± 20.49 ***	462.62 ± 11.75
Perimeter ^b^ (µm)	94.44 ± 1.35 **	76.97 ± 0.94
Protein Content ^a^ (µg/10^6^ cells)	490 ± 57	520 ± 52
β-Glucuronidase Content ^a^ (U/10^6^ cells) ^c^	2.90 ± 0.36	2.68 ± 0.42

^a^ (*n* = 15), ^b^ (*n* = 100), ^c^ 1 U of β-glucuronidase releases 1 µg/h of phenolphthalein from phenolphthalein glucuronide at 37 °C. ** *p* < 0.05 vs. HDMs, *** *p* < 0.001 vs. HDMs.

**Table 2:** Common surface marker expression on LDMs and HDMs *.

>85%		<5%	
CD11c	β_2_-integrin/Gp150	CD1a	Dendritic cells
CD44	Hyaluronic acid receptor/Pgp-1	CD4	T cells
CD71	Transferrin receptor	CD14	LPS coreceptor
HLA-DR	Class II MHC	CD15	X-Apten
		CD25	IL-2Rα
		CD35	C3b receptor/Monocytes
		CD38	Cyclic ADP ribose hydrolase
		CD42b	GPIbα Platelets
		CD56	NCAM NK cells
		CD61	Integrin β_3_ Platelets
		CD69	Eosinophils
		CD80	T cell costimulatory molecule
		CD117	KIT Mast cells
		CD130	IL-6Rβ
		CD154	CD40L receptor

* Percent of positive cells.

**Table 3:** Differential expression of surface molecules between LDMs and HDMs.

CD Denomination	LDMs	HDMs		
	% of Positive Cells Mean Fluorescence Intensity (MFI)			
CD40 CD-40L receptor (APC)	29.23 ± 5.14 189 ± 39		53.12 ± 3.34 312 ± 44	*p* < 0.01 *p* < 0.05
CD45 Leucocyte common antigen (leucocytes)	67.41 ± 7.36 412 ± 82		94.31 ± 2.03 640 ± 99	*p* < 0.01 *p* < 0.05
CD63 Type III glycoprotein (activated macrophages)	65.21 ± 6.26 373 ± 29		43.65 ± 4.18 192 ± 18	*p* < 0.05 *p* < 0.01
CD64 Fcγ-RI (monocytes/macrophages)	50.62 ± 7.54 272 ± 28		35.33 ± 7.42 161 ± 20	*p* < 0.05 *p* < 0.01
CD86 Costimulation molecule (APC)	55.41 ± 6.32 305 ± 38		81.21 ± 6.13 662 ± 95	*p* < 0.05 *p* < 0.05

## Data Availability

The data presented in this study are available on request from the corresponding authors.

## Supplementary Materials

The following are available online at https://www.mdpi.com/article/10.3390/biomedicines9050505/s1, Figure S1: Gating strategy used to identify monocytes and lymphocytes in human lung macrophage preparations, Figure S2: Gating strategy used to identify monocytes in human lung macrophage preparations, Figure S3: Gating strategy used to identify granulocytes and monocytes in human lung macrophage preparations.

## Abbreviations

Abs	antibodies
APC	antigenic-presenting cell
BAL	bronchoalveolar lavage
BSA	bovine serum albumin
HDM	high-density macrophage
LDM	low-density macrophage
LDN	low-density neutrophils
LPS	lipopolysaccharide
NDN	normal-density neutrophil
PBS	phosphate-buffered saline
PIPES	piperazine-1,4-bis (2-ethanesulfonic acid)
TLR4	toll-like receptor 4
