# Supplementary material for: Phenotypic and Functional Heterogeneity of Low-Density and High-Density Human Lung Macrophages
Source: Biomedicines. 2021 May 4;9(5):505. doi: 10.3390/biomedicines9050505 (PMC8147777; doi:10.3390/biomedicines9050505)
Supplement: Supplementary file 1 [file biomedicines-09-00505-s001.zip › biomedicines-1076656-supplementary.pdf]

Supplementary

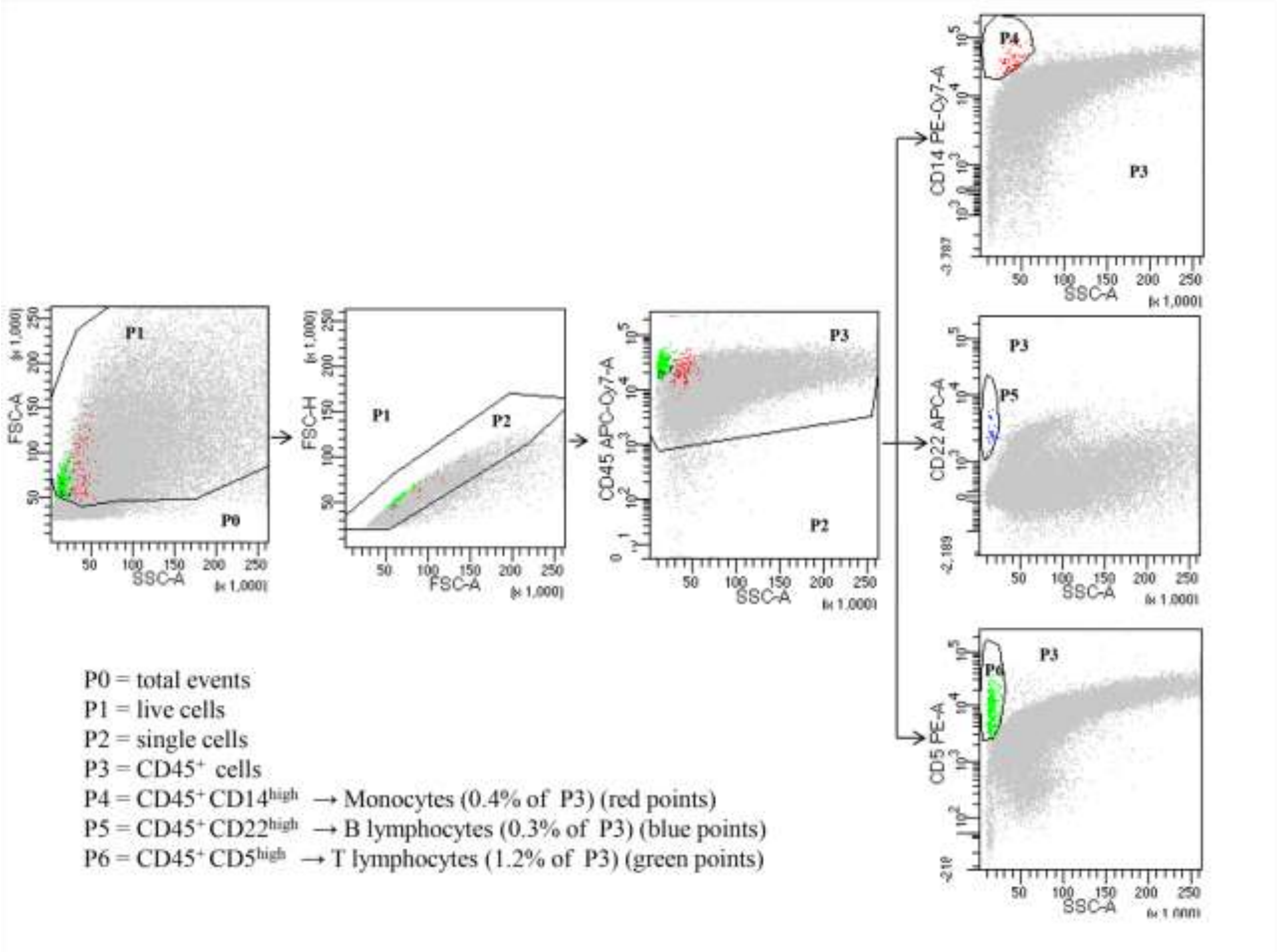

Supplementary Figure 1. Representative flow cytometric panels with respect to the gating strategy used to identify monocytes and lymphocytes in human lung macrophage preparations. Among total cells (P0), after excluding dead cells (P1) and doublets (P2), leukocytes were identified as CD45^{+} (P3). Among CD45^{+} cells (P3), monocytes were identified as CD45^{+}CD14^{high} (P4), B lymphocytes were identified as CD45^{+}CD22^{+} (P5) and T lymphocytes were identified as CD45^{+}CD5^{+} (P6). Results are representative of three independent experiments.

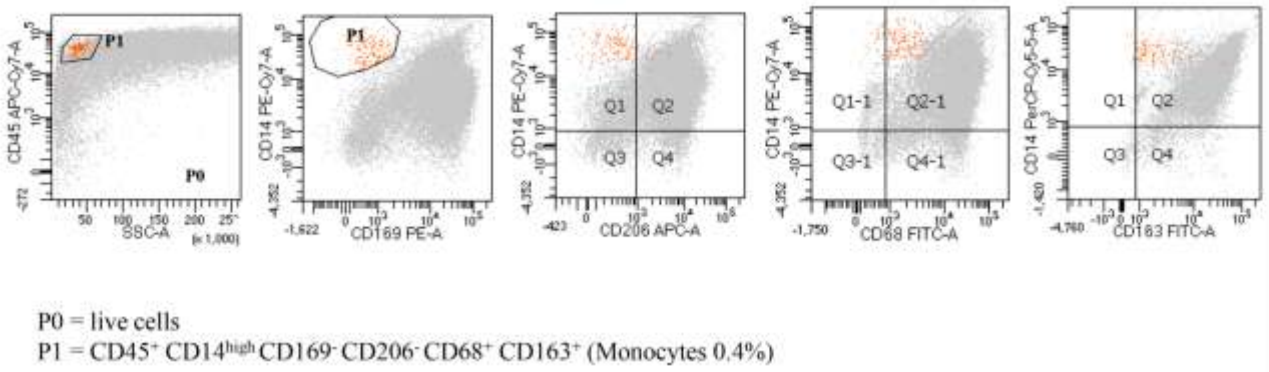

Supplementary Figure 2. Representative flow cytometric panels with respect to the gating strategy used to identify monocytes in human lung macrophage preparations. Among live cells (P0), monocytes were identified as $CD45^{+}$ $CD14^{high}$ $CD169^{-}$ $CD206^{-}$ $CD68^{+}$ $CD163^{+}$. Results are representative of three independent experiments.

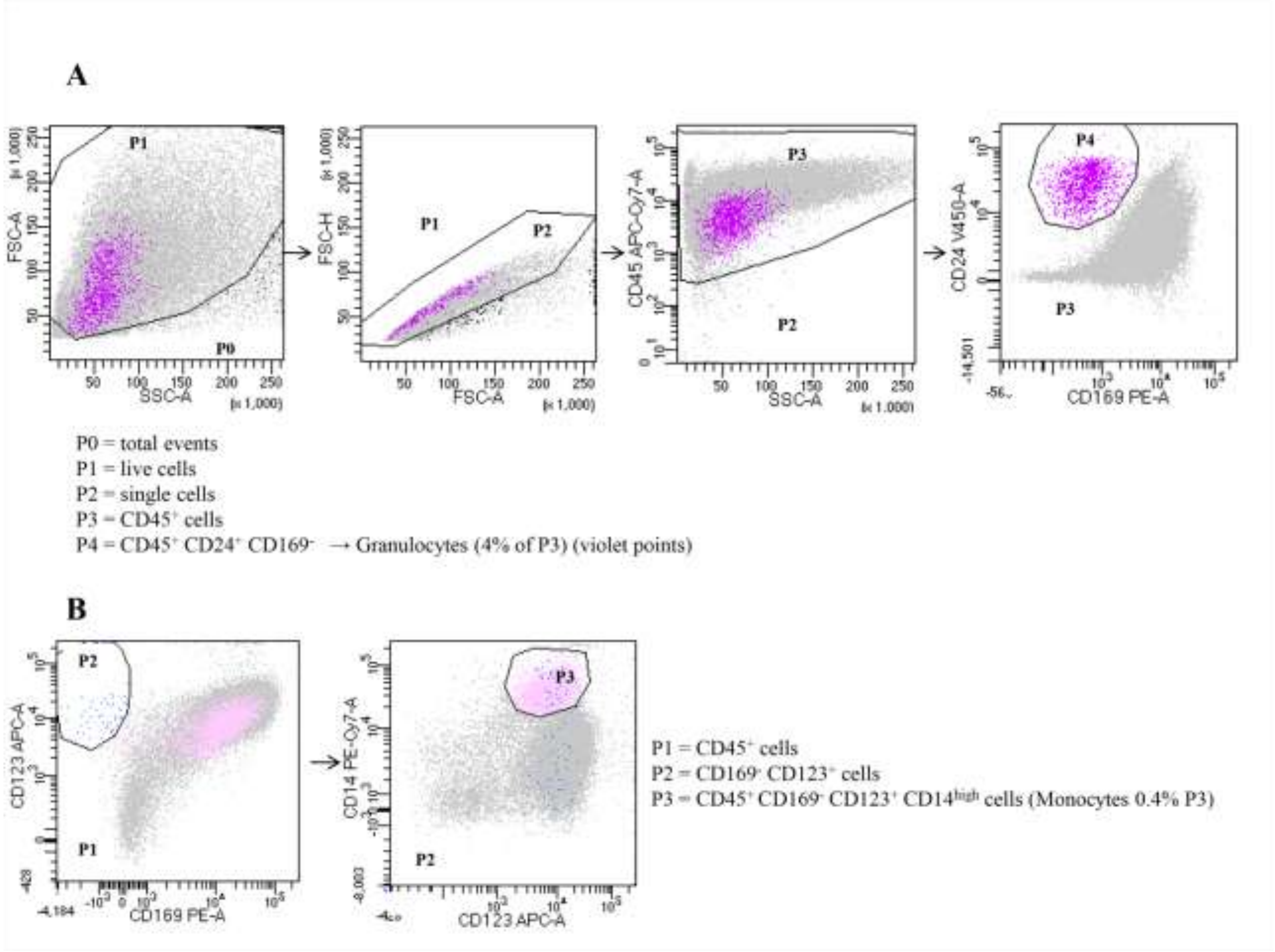

Supplementary Figure 3. Representative flow cytometric panels with respect to the gating strategy used to identify granulocytes and monocytes in human lung macrophage preparations. Among total cells (P0), after excluding dead cells (P1) and doublets (P2), leukocytes were identified as $CD45^+$ (P3). (Panel A) Among $CD45^+$ cells (P3), granulocytes were identified as $CD45^+CD169^-CD24^{high}$ (P4). (Panel B) Monocytes were identified as $CD45^+$ (P1) $CD169^-$ $CD123^+$ (P2) $CD14^{high}$ (P3). Results are representative of three independent experiments.
